# Ion-Induced Nanoscale Ripple Patterns on Si Surfaces: Theory and Experiment

**DOI:** 10.3390/ma3104811

**Published:** 2010-10-22

**Authors:** Adrian Keller, Stefan Facsko

**Affiliations:** 1 Institute of Ion Beam Physics and Materials Research, Forschungszentrum Dresden-Rossendorf, P.O. Box 51 01 19, D-01314 Dresden, Germany; 2 Interdisciplinary Nanoscience Center (iNANO), Aarhus University, Ny Munkegade, DK-8000 Aarhus C, Denmark

**Keywords:** nanopatterning, ion sputtering, surface morphology, continuum theory

## Abstract

Nanopatterning of solid surfaces by low-energy ion bombardment has received considerable interest in recent years. This interest was partially motivated by promising applications of nanopatterned substrates in the production of functional surfaces. Especially nanoscale ripple patterns on Si surfaces have attracted attention both from a fundamental and an application related point of view. This paper summarizes the theoretical basics of ion-induced pattern formation and compares the predictions of various continuum models to experimental observations with special emphasis on the morphology development of Si surfaces during sub-keV ion sputtering.

## 1. Introduction

Back in the 1960s, Navez *et al.* studied the morphology of glass surfaces bombarded with a 4 keV ion beam of air [1]. During the sputtering, they found the surface to develop periodic structures with lateral dimensions ranging from 30 to 120 nm depending on the angle of incidence. The orientation of the structures was determined by the direction of the ion beam. For grazing incidence, ripple patterns oriented parallel to the projection of the ion beam were observed whereas the ripples were rotated by 90${}^{\circ }$ at near-normal incidence. At normal incidence, however, the surface developed dot-like features. In the following years, sputter-induced ripple structures were found on all kinds of amorphous as well as crystalline materials like insulators [2], semiconductors [2,3], and metals [4].

During the 1990s, several *in-situ* and *ex-situ* studies investigated the ion-induced formation of nanoripples by means of new techniques for the exact characterization of the eroded surfaces like light scattering [5] and x-ray methods [6], as well as scanning tunneling [7] and atomic force microscopy [8,9]. In 1999, Facsko *et al.* observed the formation of hexagonally ordered nanodots on GaSb surfaces during normal incidence ion sputtering [10]. Such regular dot patterns have been found on various semiconductor surfaces sputtered at normal incidence [11] as well as off-normal incidence with [12] and without sample rotation [13].

Nowadays, ion-induced nanopatterns become interesting for various technological applications. Recent experiments demonstrate the principal applicability of nanoripples in the fabrication of microelectronic devices [14] and optically active nanostructure arrays [15,16]. Another approach uses nanodot formation under normal incidence sputtering of layer stacks to create isolated magnetic islands for magnetic storage media [17,18]. In addition, rippled substrates are becoming popular as templates for thin film deposition. It was shown that the morphology of the nanorippled substrates modifies the magnetic properties of ultrathin single-crystalline [19] and poly-crystalline [20,21,22] metal films. In a similar manner, arrays of close-packed nanomagnets could recently be obtained by shadow deposition on hexagonally ordered dot patterns [23]. Moreover, the self-organized alignment of physical-vapor deposited metal nanoparticles on nanorippled substrates was recently observed, leading to large arrays of nanoparticle chains exhibiting polarization-dependent plasmon absorption [24,25]. With the same technique, also arrays of metallic nanowires could be produced [26,27,28]. Most of these applications crucially depend on certain properties of the template patterns such as a high degree of order in the case of storage media [17,23] or a well defined ripple wavelength that fits to the growth conditions of the nanoparticles [24]. A precise control of the pattern properties in turn requires detailed knowledge of the pattern formation process and the contributing mechanisms. Up to now, however, this knowledge is still incomplete.

Although several possible origins of the ripple patterns like ion-induced local stresses or initial surface defects have been suggested in the years following their discovery [3], no conclusive explanation could be found until 1988. In this year, Bradley and Harper developed a continuum model [29] to describe the formation of the ripple patterns based on the so-called micro-roughening instability [30]. It was already shown by Sigmund [30] that the local erosion rate of a surface under ion bombardment is higher in depressions than on elevations. This curvature dependence of the sputter yield induces an instability of the surface against periodic disturbances which leads to an amplification of all initial modulations. In the presence of a competing smoothing process like surface self-diffusion, however, a wavelength selection is observed with the most unstable mode growing fastest [29].

The resulting linear continuum equation, the so-called Bradley-Harper (BH) equation, is able to reproduce some of the main experimentally observed features of the formation and early evolution of the patterns like their orientation with respect to the ion beam and the exponential growth of the ripple amplitude. For long sputtering times, however, certain experimental observations such as the saturation of the ripple amplitude cannot be explained within the framework of the linear model. This disagreement was attributed to a growing influence of nonlinear terms that dominate the morphology at later times. Hence, in 1995, Cuerno and Barabási derived a nonlinear continuum equation of the Kuramoto-Sivashinsky (KS) [31,32] type to describe the ion-induced formation of periodic surface structures [33]. In the early time regime, this equation behaves like the linear BH equation. At a certain transition time, however, the nonlinear terms start to control the evolution of the surface [34]. When entering this nonlinear regime, the amplitude of the ripples saturates as found experimentally. However, a transition to kinetic roughening with a loss of lateral order is observed in this regime [34,35]. Whereas such a transition has been observed in a few experiments [36], other studies report a stabilization of the regular patterns at high fluences [37,38,39]. Another feature of the experimental pattern evolution that could not be reproduced by the KS equation is the occasionally observed coarsening of the pattern wavelength [9,11,12,39,40,41,42,43,44]. In order to overcome these discrepancies, several other nonlinear models based on the KS equation have been proposed [45,46,47,48,49]. These models all show a similar behavior in their linear regime and make different predictions only for the surface evolution in the nonlinear regime corresponding to rather long sputter times [50]. Therefore, a distinct demand for high fluence experiments has evolved which investigate the evolution of the surface morphology in the nonlinear regime in order to identify the continuum model that describes the given experimental system.

In the following section, the theoretical basics of ion-induced pattern formation are summarized and the various continuum equations available at present are discussed. Section 3 shows experimental results on the pattern formation and evolution on Si surfaces and tries to identify a certain continuum equation to describe the surface evolution. In addition, dependencies on experimental parameters are discussed with respect to possible applications. Section 4 provides a summary.

## 2. Continuum Theory of Ripple Formation During Low Energy Ion Sputtering

If a solid surface is bombarded with energetic ions, surface material will be removed [51,52]. The theoretical description of this mechanisms called *sputtering* has already been formulated in the 1960s by Sigmund [53]. The ions penetrating into the target surface are slowed down and lose their kinetic energy and momentum in elastic and inelastic collisions with target nuclei and electrons, respectively. For kinetic energies of the order of some keV and below, however, the momentum and kinetic energy of the ions are transferred to the target atoms in nuclear collisions mainly and inelastic collisions play only a minor role [54]. A target atom taking part in one of these collisions receives some of the ion’s kinetic energy and momentum and can, therefore, be set in motion. If such an atom obtains sufficient energy, it can induce further collisions with other target atoms, thus increasing the number of moving atoms. This situation is then called collision cascade [54]. For typical ion fluxes, the collision cascades do not overlap in space and time and can therefore be treated independently. Within one collision cascade, it may happen that a target atom receives momentum directed towards the surface. If the kinetic energy of such an atom is high enough to overcome the surface binding energy, it will leave the surface and be sputtered away. Under continuous irradiation, the surface will be eroded as a whole. Additional effects that also might cause the removal of target material such as the deposition of potential energy during the impact of slow multiply-charged ions [55] will not be treated in this review.

When bombarding a crystalline non-metallic surface, e.g., a semiconductor, one can observe an additional effect. The number of generated defects in the crystal increases with the number of ion impacts. Therefore, for a large number of ion impacts, the crystal structure of the surface becomes unstable and the whole surface gets amorphized [54]. For single crystalline Si surfaces bombarded at energies of a few hundred eV at room temperature, this amorphisation is observed already after the impact of about $10^{15}$ ions per cm${}^{2}$ [56]. For higher fluences, the surface can be treated as fully amorphous.

### 2.1. Sigmund’s theory of sputtering

A keV ion penetrating a solid surface loses its kinetic energy mainly in nuclear collisions with target atoms. The energy loss per unit path length, or stopping power, is then given by

(1) $$\frac{dE}{dz}=-NS_{n}\left(E\right)$$

with the atomic density *N* of the solid and the nuclear stopping cross section $S_{n}\left(E\right)$. *E* is the initial kinetic energy of the penetrating ion.

The nuclear stopping cross section $S_{n}\left(E\right)$ depends on the interaction potential used to model the collision between ion and target atom. With the power approximation of the Thomas-Fermi potential as a common choice, $S_{n}\left(E\right)$ reads [53]

(2) $$S_{n}\left(E\right)=\frac{1}{1-m}C_{m}\omega^{1-m}E^{1-2m}.$$

Here, *m* accounts for the Coulomb screening of the nuclei due to the electrons in the solid and ranges from 0 to 1. In the lower-keV and upper eV region, m=1/3 is commonly assumed, whereas *m* should be close to zero in the eV region [53]. $C_{m}$ and *ω* are constants that incorporate the atomic parameters of the projectile and target species:

(3) $$\begin{array}{ccc} C_{m} & = & \frac{\pi }{2}\lambda_{m}a_{TF}^{2}{\left(\frac{M_{p}}{M_{t}}\right)}^{m}{\left(\frac{2Z_{p}Z_{t}e^{2}}{a_{TF}}\right)}^{2m},\\ \omega & = & \frac{4M_{p}M_{t}}{{\left(M_{p}+M_{t}\right)}^{2}}. \end{array}$$

$M_{p,t}$ is the atomic mass and $Z_{p,t}$ the atomic number of the projectile and the target atom, respectively. $\lambda_{m}$ is a dimensionless function of *m* with values ranging from $\lambda_{1}=0.5$ to $\lambda_{0}∼24$ and $a_{TF}$ is the Thomas-Fermi screening length.

The average number of sputtered atoms per incident ion is given by the sputtering yield *Y*. For linear collision cascades, *i.e.,* for a sufficiently small number and isotropic distribution of binary collisions within one cascade [54], the sputtering yield *Y* is proportional to the energy $F_{D}\left(z\right)$ deposited per unit depth in the surface at z=h by a certain ion at the lateral position (x,y),

(4) $$Y\left(E,\theta ,x,y\right)=\Lambda F_{D}\left(E,\theta ,x,y,z=h\right)$$

with the ion energy *E* and the angle of incidence *θ*. Λ is given by

(5) $$\Lambda =\frac{\Gamma_{m}}{8(1-2m)}\frac{1}{NC_{m}E_{sb}^{1-2m}}.$$

Here, $E_{sb}$ is the surface binding energy and $\Gamma_{m}$ a function of *m* given by

(6) $$\Gamma_{m}=\frac{m}{\frac{d}{dx}\left[\ln\Gamma \left(x=1\right)\right]-\frac{d}{dx}\left[\ln\Gamma \left(x=1-m\right)\right]}.$$

Because the majority of the sputtered particles originates from secondary collisions with low energy (< 50 eV) recoils, Sigmund suggested m=0 for Equation (5) [53], resulting in $\Gamma_{0}=6/\pi^{2}$. Therefore, Equation (5) becomes

(7) $$\Lambda =\frac{3}{4\pi^{2}}\frac{1}{NC_{0}E_{sb}},$$

with $C_{0}=0.0181$ nm${}^{2}$ [53].

For a plane and homogeneous surface, the deposited energy does not depend on the lateral position of the ion impact and is given by

(8) $$F_{D}\left(E,\theta \right)=\alpha NS_{n}\left(E\right),$$

with *α* being a dimensionless function of the angle of incidence *θ* and the mass ratio $M_{t}/M_{p}$ [53]. Then, the sputtering yield becomes

(9) $$Y\left(E,\theta \right)=\frac{4.2}{{\mathrm{nm}}^{2}}\frac{\alpha S_{n}\left(E\right)}{E_{sb}}.$$

According to Equation (9), the sputter yield depends on the surface binding energy and due to Equations (2) and (3) also on the atomic species. Therefore, for a multicomponent material, different sputter yields for individual atomic species *i* might be observed. In a first approximation, the total sputter yield can be treated as the sum of the different components according to their surface concentration. For this purpose, so-called “component” sputtering yields $Y_{i}^{c}$ are defined such that the partial sputtering yields $Y_{i}$ follow the relation

(10) $$Y_{i}=q_{i}^{s}Y_{i}^{c}$$

with the surface atomic fractions $q_{i}^{s}$. Then, the total sputtering yield is given by

(11) $$Y=\sum_{i}Y_{i}.$$

Different component sputtering yields then lead to

(12) $$\frac{Y_{i}}{Y_{j}}\neq \frac{q_{i}^{s}}{q_{j}^{s}},$$

*i.e.*, one or more components are sputtered preferentially. Due to this preferential sputtering, the surface concentrations are altered at increasing fluence even in a homogeneous material. For a two-component material with the components *A* and *B*, preferential sputtering of *A* leads to a decrease of the surface concentration and thus also to a decrease of the partial sputtering yield of *A*. Prolonged sputtering will then lead to a stationary state described by

(13) $$\frac{Y_{A}^{\infty }}{Y_{B}^{\infty }}=\frac{q_{A}}{q_{B}}$$

which is characterized by the stationary partial sputtering yields $Y_{i}^{\infty }$ and the bulk atomic fractions $q_{i}$. In the stationary state, the altered composition profiles remain constant but are moved into the bulk due to sputter erosion, so that atoms sputtered at the surface must be balanced by atoms fed from the bulk into the altered surface layer. From Equation (10) and (13),

(14) $$\frac{{\left(q_{A}^{s}\right)}^{\infty }}{{\left(q_{B}^{s}\right)}^{\infty }}={\left(\frac{q_{A}}{q_{B}}\right)}^{2}\frac{Y_{B}^{0}}{Y_{A}^{0}}$$

for the stationary surface composition is obtained with the stationary surface atomic fractions ${\left(q_{i}^{s}\right)}^{\infty }$ and the initial partial sputtering yields $Y_{i}^{0}$.

### 2.2. The Bradley-Harper model

If a surface is bombarded with a homogeneous flux of ions *j*, then the over-all energy deposited in a given point *A* of the surface is the sum of the energy deposited in this point due to all surrounding ion impacts. Therefore, with Equation (4), the local erosion rate in point *A* is given by the integral over all contributing events [30]

(15) $$v\left(A\right)=\frac{\Lambda }{N}\int \int \phi \left(r\right)E_{D}\left(r\right)dxdy$$

where ϕ(r) is the flux of incoming ions *j* corrected for the local angle of incidence and $E_{D}\left(r\right)$ is the energy deposited per unit volume at r=(x,y,z). $E_{D}\left(r\right)$ is related to $F_{D}\left(z\right)$ of Equation (4) by [30]

(16) $$F_{D}\left(z\right)=\int \int E_{D}\left(r\right)dxdy.$$

The spatial distribution of the deposited energy $E_{D}\left(r\right)$ can be approximated by a Gaussian,

(17) $$E_{D}\left(r\right)=\frac{E}{{\left(2\pi \right)}^{3/2}\sigma \mu^{2}}\exp\left(-\frac{{\left(z+a\right)}^{2}}{2\sigma^{2}}-\frac{x^{2}+y^{2}}{2\mu^{2}}\right).$$

**Figure 1:**
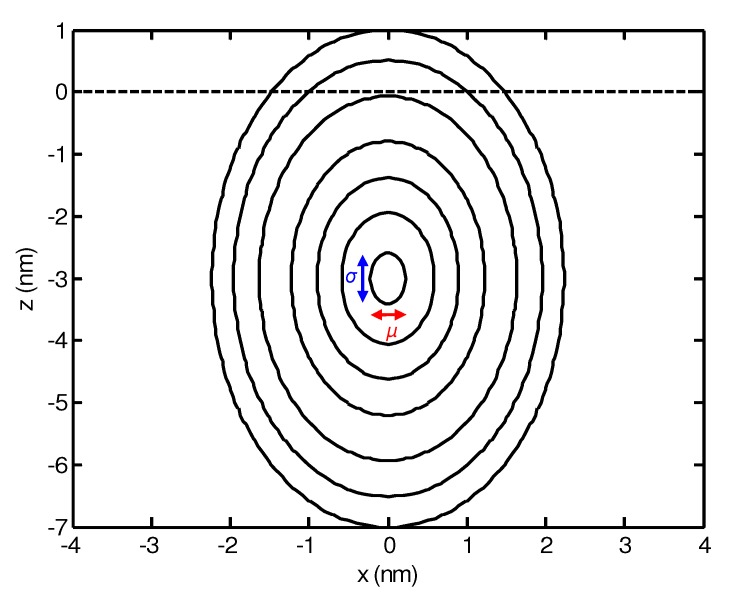
Contour plot of the deposited energy in the *x*-*z* plane according to equation (17) with a=3 nm, σ=0.9 nm, μ=0.5 nm, and E=500 eV. The surface at z=0 is indicated by the broken line.

Here, *μ* and *σ* represent the lateral and longitudinal width of the distribution, respectively, and *a* is the mean penetration depth of the ion. A contour plot of the energy distribution is shown in Figure 1.

**Figure 2:**
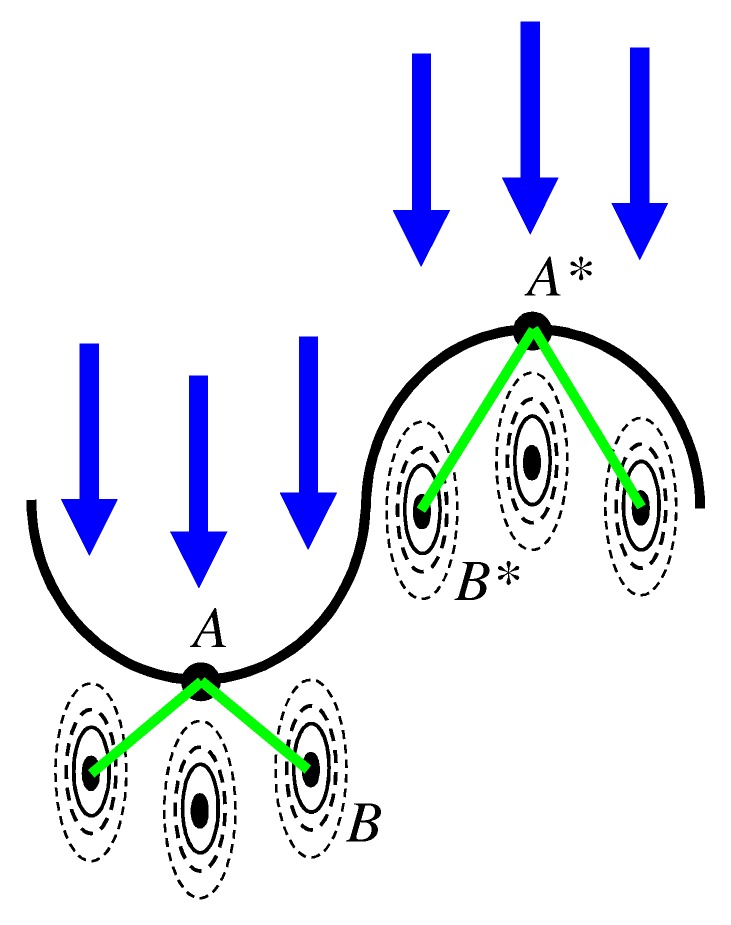
Schematic drawing of the energy deposition in rough surfaces, see text.

For a rough surface sputtered with an uniform flux of ions, the energy deposited in the surface is not constant but rather depends on the lateral position r. To some extent, this is caused by the angular dependence of the ion flux at the surface. In addition, however, the energy deposition into the surface depends on the local shape of the surface. This lateral variation of the energy deposition causes a lateral variation of the local erosion rate and, therefore, a change of the surface morphology with sputtering time [30]. A closer inspection of the underlying mechanisms reveals that the local erosion rate is higher in troughs than on crests. This is demonstrated in Figure 2 where ions penetrate into a surface region with positive (Figure 2 left) and negative (Figure 2 right) curvature, respectively. The Gaussian distribution of the deposited energy is centered at the mean penetration depth *a* of the ions and indicated by the (broken) lines of constant energy. From Figure 2 it is obvious that the distance from the surface point *A* where the sputtering occurs to the contributing impact at *B* is shorter than the distance $A^{*}-B^{*}$. Therefore, the over-all deposited energy and also the erosion rate is larger in points with positive curvature (*A*) than in those with negative curvature ($A^{*}$). Obviously, the surface becomes unstable and the initial surface roughness gets amplified. This mechanism is called *surface micro-roughening* [30].

In order to explain the formation of periodic ripple patterns during sputtering, Bradley and Harper have calculated the integral (15) under the assumption of large radii of curvature $R_{x}$ and $R_{y}$ [29]. Then, the time evolution of the continuous surface height function h(x,y,t) is given by

(18) $$\frac{\partial h}{\partial t}=-v\left(\varphi ,R_{x},R_{y}\right)\sqrt{1+{\left(\nabla h\right)}^{2}}$$

with *φ* being the angle between the direction of the ion beam and the local surface normal [33]. The projected direction of the ion beam is parallel to the *x* axis. Equation (18) can then be expanded in terms of derivatives of the surface height [33]. To first order in the surface curvature, Bradley and Harper obtained

(19) $$\frac{\partial h}{\partial t}=-v_{0}+\gamma \frac{\partial h}{\partial x}+\nu_{x}\frac{\partial^{2}h}{\partial x^{2}}+\nu_{y}\frac{\partial^{2}h}{\partial y^{2}}.$$

Here, $v_{0}$ is the erosion velocity of the planar surface, *γ* causes a lateral movement of the structures, and the micro-roughening instability is incorporated by the coefficients $\nu_{x,y}$. These coefficients are given by the following relations [45]:

(20) $$\begin{array}{ccc} v_{0} & = & Fc, \end{array}$$

(21) $$\begin{array}{ccc} \gamma & = & F\frac{s}{f^{2}}\left[a_{\sigma }^{2}a_{\mu }^{2}c^{2}\left(a_{\sigma }^{2}-1\right)-a_{\sigma }^{4}s^{2}\right], \end{array}$$

(22) $$\begin{array}{ccc} \nu_{x} & = & Fa\frac{a_{\sigma }^{2}}{2f^{3}}\left[2a_{\sigma }^{4}s^{4}-a_{\sigma }^{4}a_{\mu }^{2}s^{2}c^{2}+a_{\sigma }^{2}a_{\mu }^{2}s^{2}c^{2}+a_{\mu }^{4}c^{4}\right], \end{array}$$

(23) $$\begin{array}{ccc} \nu_{y} & = & -Fa\frac{c^{2}a_{\sigma }^{2}}{2f}, \end{array}$$

with

(24) $$\begin{array}{ccc} F & = & \frac{jE\Lambda a}{\sigma \mu N\sqrt{2\pi f}}e^{-a_{\sigma }^{2}a_{\mu }^{2}c^{2}/2f},\\ f & = & a_{\sigma }^{2}s^{2}+a_{\mu }^{2}c^{2},\\ a_{\sigma }=\frac{a}{\sigma } & , & a_{\mu }=\frac{a}{\mu },\\ s=\sin\theta & , & c=\cos\theta . \end{array}$$

When sputtering a surface at finite temperature, atoms will diffuse on the surface leading to a relaxation of the surface. This effect, the so-called Herring-Mullins surface diffusion [57,58], can be introduced by adding a term proportional to the fourth derivative of the surface height to Equation (19), resulting in [29]

(25) $$\frac{\partial h}{\partial t}=-v_{0}+\gamma \frac{\partial h}{\partial x}+\nu_{x}\frac{\partial^{2}h}{\partial x^{2}}+\nu_{y}\frac{\partial^{2}h}{\partial y^{2}}-K\nabla^{4}h.$$

In the Bradley-Harper (BH) equation (25), *K* is the relaxation rate due to thermally activated surface self-diffusion [29],

(26) $$K=\frac{D_{s}\varrho n_{d}}{N^{2}k_{B}T},$$

with the surface self-diffusivity $D_{s}$, the surface free energy per unit area *ϱ*, the areal density of diffusing atoms $n_{d}$, the Boltzmann constant $k_{B}$ and the temperature *T*.

The behavior of Equation (25) shall be analyzed by calculating its Fourier transform. Be $\tilde{h}\left(k,t\right)$ the Fourier transform of the surface height function h(r,t) with the wave vector $k=k_{x}e_{x}+k_{y}e_{y}$ and r=(x,y). Then, Equation (25) can be written as

(27) $$\frac{\partial \tilde{h}\left(k,t\right)}{\partial t}=\left[-\left(\nu_{x}k_{x}^{2}+\nu_{y}k_{y}^{2}\right)-K{\left(k_{x}^{2}+k_{y}^{2}\right)}^{2}\right]\tilde{h}\left(k,t\right).$$

Integration of Equation (27) yields

(28) $$\tilde{h}\left(k,t\right)={\tilde{h}}_{0}\left(k\right)\exp\left(R_{k}t\right),$$

with the growth rate $R_{k}=-\left[\nu_{x}k_{x}^{2}+\nu_{y}k_{y}^{2}+K{\left(k_{x}^{2}+k_{y}^{2}\right)}^{2}\right]$. Therefore, spatial frequencies k with positive $R_{k}$ grow exponentially in amplitude, whereas those with negative $R_{k}$ decay exponentially with time. Because of the positive value of *K*, surface roughening occurs only for negative $\nu_{x,y}$. The maximum value of $R_{k}$ is reached for

(29) $$k_{c}=\sqrt{\frac{|\min\left(\nu_{x},\nu_{y}\right)|}{2K}}.$$

Therefore, the Fourier component of the initial roughness spectrum with the wave number $k_{c}$ will grow fastest, resulting in a wavelike surface pattern with a periodicity

(30) $$\lambda =\frac{2\pi }{k_{c}}=2\pi \sqrt{\frac{2K}{|\min\left(\nu_{x},\nu_{y}\right)|}}.$$

**Figure 3:**
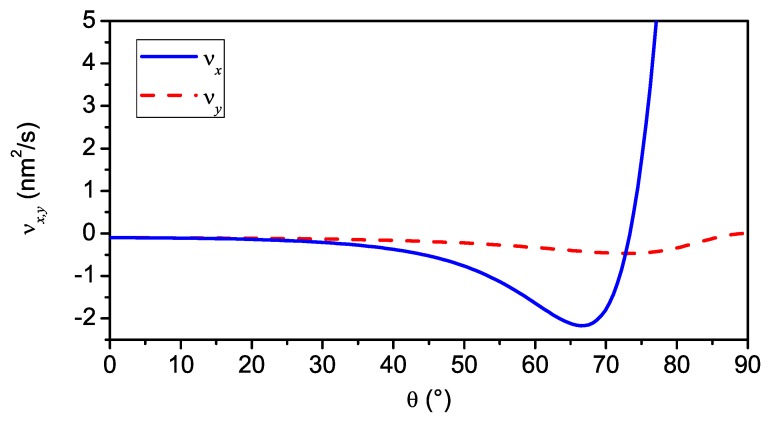
$\nu_{x,y}$ *versus* angle of incidence *θ*, calculated for 500 eV Ar${}^{+}$ irradiation of Si with $j=1\times 10^{15}$ cm${}^{-2}$ s${}^{-1}$, a=2 nm, σ=0.8 nm, μ=0.4 nm, and Y=2.

**Figure 4:**
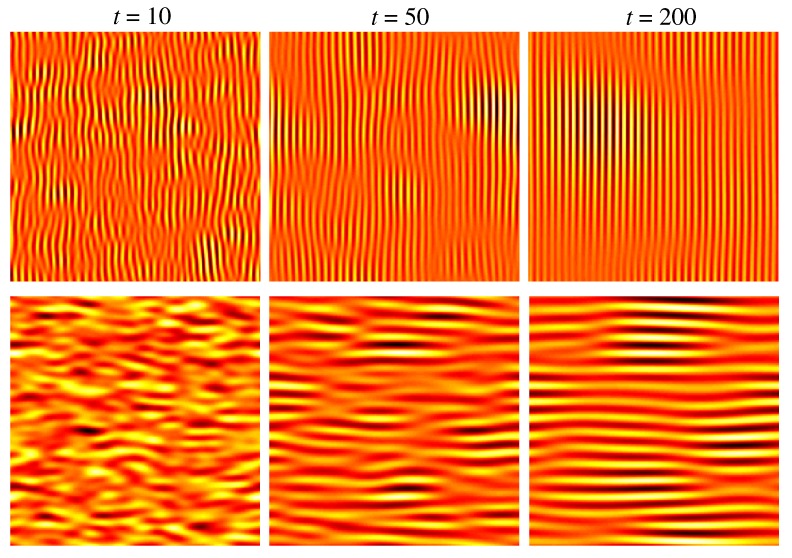
Numerical integration of the BH equation (25) for the same parameters as in Figure 3 at $\theta =65^{\circ }$ (upper row) and $\theta =75^{\circ }$ (lower row) and different times *t*. *K* was set to K=1.

For $\nu_{x}<\nu_{y}$ and $\nu_{x}>\nu_{y}$, the wave vector of the observed pattern is $k_{c}=k_{c}e_{x}$ and $k_{c}=k_{c}e_{y}$, respectively. The angular dependence of $\nu_{x,y}$ for a certain set of microscopic parameters is shown in Figure 3. At an angle of $\theta ∼73^{\circ }$, one observes a change from $\nu_{x}<\nu_{y}$ to $\nu_{x}>\nu_{y}$ what corresponds to a rotation of the observed ripple pattern from normal to parallel with respect to the projected direction of the ion beam. This is demonstrated in Figure 4 which depicts numerical integrations [59] of Equation (25) at $\theta =65^{\circ }$ (upper row) and $\theta =75^{\circ }$ (lower row) at different times *t*. This type of pattern rotation with increasing incident angle has been observed in several experiments [1,36,40,44,60,61,62]. Some other predictions of the BH equation, however, are at variance with certain experimental observations:

- The amplitude of the ripples should grow exponentially without saturation. In experiments, however, saturation of the ripple amplitude at a constant value is observed after an initial exponential increase [63,64].
- From equations (30), (22), and (23) it follows that $\lambda \propto j^{-1/2}$. In contrast, some experimental studies report the ripple wavelength *λ* to be constant with the ion flux *j* [65].

- Furthermore, from the same equations *λ* follows to be a function of the ion energy *E* and the penetration depth *a*, which again is a function of *E*. Therefore, one expects the ripple wavelength to decrease with the ion energy as $\lambda \propto E^{p}$ with the negative exponent *p* [66]. However, this behavior is in general only observed at relatively high temperatures [67]. At low and moderate temperatures, several studies report the ripple wavelength to increase with energy [38,39,44,68,69].
- Equations (30) and (26) indicate a dependence of *λ* on the sample temperature. However, in the case of GaAs and InP, such a dependence of the wavelength was only observed at elevated temperatures whereas *λ* was found to be constant at room temperature and below [70]. Another study on SiO${}_{2}$ surfaces found *λ* to be relatively constant with temperature even up to about $200^{\circ }$C [71].
- Equation (30) implies that the ripple wavelength *λ* is independent on the ion fluence Φ and should, therefore, be constant with sputtering time. Several experiments, however, show an increase of *λ* with fluence [9,39,40,41,42,43,44,72]. This phenomenon is usually referred to as *wavelength coarsening*.

Several attempts have been made in order to overcome these deficiencies of the BH equation and shall be discussed in the following.

### 2.3. Nonlinear continuum equations

#### 2.3.1. Kuramoto-Sivashinsky equation

In the series expansion of Equation (18), Bradley and Harper considered only linear terms. Cuerno and Barabási, however, took the expansion to lowest nonlinear order resulting in [33]

(31) $$\begin{array}{ccc} \frac{\partial h}{\partial t} & = & -v_{0}+\gamma \frac{\partial h}{\partial x}+\nu_{x}\frac{\partial^{2}h}{\partial x^{2}}+\nu_{y}\frac{\partial^{2}h}{\partial y^{2}}\\ & & +\frac{\zeta_{x}}{2}{\left(\frac{\partial h}{\partial x}\right)}^{2}+\frac{\zeta_{y}}{2}{\left(\frac{\partial h}{\partial y}\right)}^{2}-K\nabla^{4}h+\eta . \end{array}$$

The additional nonlinear terms in this equation are non-conserved Kardar-Parisi-Zhang (KPZ) nonlinearities [73,74] that incorporate the dependence of the local erosion velocity on the absolute value of the surface slopes. Their coefficients are given by [45]

(32) $$\begin{array}{ccc} \zeta_{x} & = & F\frac{c}{2f^{4}}\left[a_{\sigma }^{8}a_{\mu }^{2}s^{4}\left(3+2c^{2}\right)+4a_{\sigma }^{6}a_{\mu }^{4}s^{2}c^{2}-a_{\sigma }^{4}a_{\mu }^{6}c^{4}\left(1+2s^{2}\right)\right.\\ & & \left.-f^{2}\left(2a_{\sigma }^{4}s^{2}-a_{\sigma }^{2}a_{\mu }^{2}\left(1+2s^{2}\right)\right)-a_{\sigma }^{8}a_{\mu }^{4}s^{2}c^{2}-f^{4}\right], \end{array}$$

(33) $$\begin{array}{ccc} \zeta_{y} & = & F\frac{c}{2f^{2}}\left[a_{\sigma }^{4}s^{4}+a_{\sigma }^{2}a_{\mu }^{2}c^{2}-a_{\sigma }^{4}a_{\mu }^{2}c^{2}-f^{2}\right]. \end{array}$$

In order to account for the stochastic arrival of the ions, the Gaussian white noise term *η*, defined as

(34) $$\langle \eta \left(r,t\right)\eta \left(r^{\prime },t^{\prime }\right)\rangle =2D_{\eta }\delta^{d}\left(r-r^{\prime }\right)\delta \left(t-t^{\prime }\right),$$

was added. Here, $D_{\eta }$ is the strength of the noise and *d* the dimension of the surface.

Equation (31) is an anisotropic stochastic generalization of the so-called Kuramoto-Sivashinsky (KS) equation which was originally proposed to describe chemical waves [31] and the propagation of flame fronts [32]. For short sputtering times, this equation behaves like the linear BH equation with an exponential increase of the ripple amplitude and constant ripple wavelength. Then, at a certain transition time

(35) $$t_{c}\propto \frac{K}{\nu_{x,y}^{2}}\ln\left(\frac{\nu_{x,y}}{\zeta_{x,y}}\right),$$

the surface enters a nonlinear regime and a saturation of the ripple amplitude as in the experiments is observed [34]. However, numerical analyses of the noisy KS equation in 1+1 and 2+1 dimensions show that the saturation of the ripple amplitude is accompanied by a transition to kinetic roughening [34,35]. In this regime, the surface does not exhibit any lateral order. Although such a transition has been observed in few experiments [36], it is at variance with several other experimental reports of a pattern conservation at high fluences [37,38,39].

#### 2.3.2. Damped Kuramoto-Sivashinsky equation

Inspired by the observation of stationary patterns in numerical simulations of the isotropic *damped* KS (dKS) equation by Paniconi and Elder [75], Facsko *et al.* adopted this equation for normal incidence ion sputtering [46]. The isotropic dKS equation is frequently used to describe different processes like compact electrodeposition growth [76] or directional solidification [75]. For oblique ion sputtering, however, the anisotropic dKS equation must be applied:

(36) $$\begin{array}{ccc} \frac{\partial h}{\partial t} & = & -v_{0}-\alpha h+\gamma \frac{\partial h}{\partial x}+\nu_{x}\frac{\partial^{2}h}{\partial x^{2}}+\nu_{y}\frac{\partial^{2}h}{\partial y^{2}}\\ & & +\frac{\zeta_{x}}{2}{\left(\frac{\partial h}{\partial x}\right)}^{2}+\frac{\zeta_{y}}{2}{\left(\frac{\partial h}{\partial y}\right)}^{2}-K\nabla^{4}h+\eta . \end{array}$$

This equation differs from the undamped KS equation (31) just by the additional damping term -αh with *α* being a damping coefficient that enters the effective growth rate of the ripple amplitude $R_{k_{c}}^{*}=R_{k_{c}}-\alpha$. This damping term induces smoothing of all spatial frequencies and, therefore, prevents kinetic roughening.

In the case of sputter erosion, the damping term in Equation (36) violates the translational invariance of the surface in the erosion direction. However, translational invariance can be restored by replacing the term -αh by $-\alpha (h-\bar{h})$ with $\bar{h}$ being the mean height of the surface and thus transforming Equation (36) into a nonlocal dKS equation [46] which again, as has been demonstrated [77], can be exactly mapped to a local dKS equation. The physical meaning of *α*, however, is still not clear in the case of sputter erosion.

The dKS equation has been extensively studied in numerical simulations [46,77,78,79,80]. It is not only able to show stationary patterns in the long-time limit but also to reproduce other features of experimental patterns like certain pattern defects [46] or the formation of structured islands [80]. However, no evidence for wavelength coarsening as observed in several experiments [9,11,12,39,40,41,42,43,44] has been found yet [79].

#### 2.3.3. General continuum equation

Although Equation (31) includes KPZ-like nonlinearities, other higher order terms are neglected [33]. The most general nonlinear equation that results from the expansion of Equation (18) is given by [45]

(37) $$\begin{array}{ccc} \frac{\partial h}{\partial t} & = & -v_{0}+\gamma \frac{\partial h}{\partial x}+\sum_{i=x,y}\left\{-\nu_{i}\frac{\partial^{2}h}{\partial i^{2}}+\zeta_{i}{\left(\frac{\partial h}{\partial i}\right)}^{2}+\Omega_{i}\frac{\partial^{2}}{\partial i^{2}}\frac{\partial }{\partial x}h+\right.\\ & & \left.\xi_{i}\left(\frac{\partial h}{\partial x}\right)\left(\frac{\partial^{2}h}{\partial i^{2}}\right)\right\}+\sum_{i,j=x,y}\left\{-D_{ij}\frac{\partial^{2}}{\partial i^{2}}\frac{\partial^{2}}{\partial j^{2}}h\right\}-K\nabla^{4}h+\eta . \end{array}$$

The coefficients of the additional linear and nonlinear terms then read

(38) $$\begin{array}{ccc} \Omega_{x} & = & -Fa^{2}\frac{3}{6f^{2}}\frac{s}{a_{\mu }^{2}}\left[f^{2}-fa_{\sigma }^{4}c^{2}-\left(a_{\mu }^{2}-a_{\sigma }^{2}\right)c^{2}\left(f+a_{\sigma }^{4}s^{2}\right)\right], \end{array}$$

(39) $$\begin{array}{ccc} \Omega_{y} & = & Fa^{2}\frac{1}{6f^{4}}\left[-3sf^{2}\left(f+a_{\sigma }^{4}s^{2}\right)+a_{\sigma }^{2}c^{2}\left(3a_{\sigma }^{2}sf+a_{\sigma }^{6}s^{3}\right)f\right.\\ & & \left.+2\left(a_{\mu }^{2}-a_{\sigma }^{2}\right)c^{2}\left(3f^{2}s+6a_{\sigma }^{4}s^{3}+a_{\sigma }^{8}s^{5}\right)\right], \end{array}$$

(40) $$\begin{array}{ccc} \xi_{x} & = & Fa\frac{a_{\sigma }^{2}sc}{2f^{5}}\left[-6a_{\sigma }^{8}s^{6}+a_{\sigma }^{8}a_{\mu }^{2}s^{4}\left(4+3c^{2}\right)-a_{\sigma }^{8}a_{\mu }^{4}c^{2}s^{4}+a_{\sigma }^{6}a_{\mu }^{4}c^{2}s^{2}\left(4-6s^{2}\right)\right.\\ & & \left.+a_{\sigma }^{6}a_{\mu }^{2}s^{4}\left(-3+15s^{2}\right)+a_{\sigma }^{4}a_{\mu }^{4}3c^{2}s^{2}\left(4+3s^{2}\right)\right.\\ & & \left.-a_{\sigma }^{4}a_{\mu }^{6}3c^{4}\left(1+s^{2}\right)+a_{\sigma }^{2}a_{\mu }^{6}c^{4}\left(9-3s^{2}\right)-3a_{\mu }^{8}c^{6}\right], \end{array}$$

(41) $$\begin{array}{ccc} \xi_{y} & = & Fa\frac{a_{\sigma }^{2}sc}{2f^{3}}\left[-a_{\sigma }^{4}a_{\mu }^{2}c^{2}+a_{\sigma }^{4}s^{2}\left(2+c^{2}\right)-a_{\mu }^{4}c^{4}+a_{\sigma }^{2}a_{\mu }^{2}c^{2}\left(3-2s^{2}\right)\right] \end{array}$$

(42) $$\begin{array}{ccc} D_{xx} & = & F\frac{a^{3}}{24}\frac{1}{f^{5}}\left[-4\left(3a_{\sigma }^{2}s^{2}f+a_{\sigma }^{6}s^{4}\right)f^{2}+a_{\sigma }^{2}c^{2}\left(3f^{2}+6a_{\sigma }^{4}s^{2}f+a_{\sigma }^{8}s^{4}\right)f\right.\\ & & \left.+2\left(a_{\mu }^{2}+a_{\sigma }^{2}\right)c^{2}\left(15a_{\sigma }^{2}s^{2}f^{2}+10a_{\sigma }^{6}s^{4}f+a_{\sigma }^{10}s^{6}\right)\right], \end{array}$$

(43) $$\begin{array}{ccc} D_{yy} & = & F\frac{a^{3}}{24}\frac{1}{f^{5}}\frac{3a_{\sigma }^{2}}{a_{\mu }^{2}}\left[f^{4}c^{4}\right], \end{array}$$

(44) $$\begin{array}{ccc} D_{xy} & = & F\frac{6a^{3}}{24}\frac{1}{f^{5}}\frac{f^{2}}{a_{\mu }^{2}}\left[-2\left(a_{\sigma }^{2}s^{2}\right)f^{2}+a_{\sigma }^{2}c^{2}\left(f^{2}+a_{\sigma }^{4}s^{2}f\right)\right.\\ & & \left.+2\left(a_{\mu }^{2}-a_{\sigma }^{2}\right)c^{2}\left(3a_{\sigma }^{2}s^{2}f+a_{\sigma }^{6}s^{4}\right)\right]. \end{array}$$

Actually, the *ξ* and Ω terms in Equation (37) have already been derived in reference [33] but were neglected since their influence on the asymptotic scaling of the surface was assumed to be of minor importance. The terms with the coefficients $D_{ij}$ enter Equation (37) in the form of diffusion-like terms proportional to the fourth derivative of the height function and thus lead to an additional *anisotropic* smoothing of the surface. Therefore, this relaxation mechanism is usually called *effective* or *ion-induced surface diffusion* (ISD) [81]. However, it is important to note that ISD results from preferential erosion during the sputtering which appears as a reorganization of the surface and does *not* involve any mass transport along the surface. Thus, ISD is strictly speaking no diffusion mechanism. This is also displayed by the fact that the coefficient $D_{xx}$ might even become negative at large incident angles, leading to an additional instability of the surface [81].

Since ISD does not depend on the temperature (cf. equations (42) - (44)), this smoothing mechanism is able to explain the temperature independence of the wavelength at low temperatures where thermal diffusion can be neglected. In this case, the ripple wavelength is given by

(45) $$\lambda_{ISD}=2\pi \sqrt{\frac{2D_{xx/yy}}{|\min\left(\nu_{x},\nu_{y}\right)|}}.$$

From Equations (22), (23), (42), (43) and (45) it follows that the wavelength at low temperatures does no longer depend on the ion flux. Moreover, with *a*, *μ*, and *σ* being proportional to $E^{2m}$ [54], we find $\lambda_{ISD}\propto E^{2m}$ and, therefore, an increase of $\lambda_{ISD}$ with the ion energy. At high temperatures, however, thermal diffusion becomes the dominating smoothing mechanism and the wavelength follows from Equation (30). Hence, with the incorporation of ISD into Equation (37), one is able to explain the experimentally observed flux and temperature independence of the wavelength, as well as its increase with ion energy. However, the fluence dependence of the ripple wavelength as observed in some experiments [9,39,40,41,42,43,44,72] still cannot be explained by the general continuum equation.

In the special case of normal ion incidence, the general continuum equation (37) is reduced to the isotropic stochastic KS equation with $\gamma =\xi_{x}=\xi_{y}=\Omega_{x}=\Omega_{y}=0$, $\nu_{x}=\nu_{y}$, $\zeta_{x}=\zeta_{y}$, and $D_{xx}=D_{yy}=D_{xy}/2$. For off-normal incidence, however, Equation (37) has a highly nonlinear character with a rich parameter space which might lead to rather complex morphologies and dynamic behaviors. Although some general features of Equation (37) have been studied [45], its detailed behavior, and especially the role of the additional nonlinearities with the coefficients $\xi_{x,y}$, is still to be investigated.

#### 2.3.4. Coupled two-field model

In order to overcome the inability of the KS-type Equations (31), (36), and (37) to predict ripple coarsening, Muñoz-García and co-workers recently developed a new nonlinear model following a hydrodynamic approach [49]. In this approach, Muñoz-García *et al.* considered two coupled fields

(46) $$\begin{array}{ccc} \frac{\partial h}{\partial t} & = & -\Gamma_{ex}+\Gamma_{ad}, \end{array}$$

(47) $$\begin{array}{ccc} \frac{\partial R}{\partial t} & = & \left(1-\phi \right)\Gamma_{ex}-\Gamma_{ad}+K\nabla^{2}R, \end{array}$$

where *h* and *R* represent the surface height function and the thickness of the mobile surface adatom layer, respectively. Here, $\bar{\phi }=\left(1-\phi \right)$ is the fraction of eroded adatoms that become mobile, $\Gamma_{ex}$ is the curvature dependent erosion rate and $\Gamma_{ad}$ is the rate of addition to the immobile bulk. $\Gamma_{ad}$ is given by

(48) $$\Gamma_{ad}=\gamma_{0}\left[R-R_{eq}\left(1-\gamma_{2x}\frac{\partial^{2}h}{\partial x^{2}}-\gamma_{2y}\frac{\partial^{2}h}{\partial y^{2}}\right)\right],$$

with the mean nucleation rate for a flat surface $\gamma_{0}$, the variation in the nucleation rate with the surface curvatures $\gamma_{2x,y}$, and the thickness of the layer of mobile atoms generated thermally without bombardment $R_{eq}$. $\Gamma_{ex}$ follows from microscopic derivations [50,82],

(49) $$\begin{array}{ccc} \Gamma_{ex} & = & \alpha_{0}\left[1+\alpha_{1x}\frac{\partial h}{\partial x}+\nabla \left(\underline{\alpha_{2}}\nabla h\right)+\frac{\partial }{\partial x}\nabla \left(\underline{\alpha_{3}}\nabla h\right)+\nabla \left(\underline{\alpha_{4}}\nabla \nabla^{2}h\right)\right.\\ & & \left.+\frac{\partial }{\partial x}h\nabla \left(\underline{\alpha_{5}}\nabla h\right)+\nabla h\left(\underline{\alpha_{6}}\nabla h\right)\right]. \end{array}$$

The coefficients $\underline{\alpha_{i}}$ of Equation (49) are 2×2 diagonal matrices, except $\underline{\alpha_{4}}=\left[\begin{array}{cc} \alpha_{4xx} & \alpha_{4xy}\\ \alpha_{4yx} & \alpha_{4yy} \end{array}\right]$. In the framework of Sigmund’s theory of sputtering, these coefficients can be related to those of the general Equation (37) so that $\alpha_{0}=v_{0}$, $\alpha_{1x}=-\gamma /v_{0}$, $\alpha_{2x,y}=-\nu_{x,y}/v_{0}$, $\alpha_{3x,y}=-\Omega_{x,y}/v_{0}$, $\alpha_{4ij}=-D_{ij}/v_{0}$, $\alpha_{5x,y}=-\xi_{x,y}/v_{0}$, and $\alpha_{6x,y}=-\zeta_{x,y}/v_{0}$.

Equations (46)-(49) can be approximated by performing a multiple scale expansion with a subsequent adiabatic elimination of *R*. This results in an equation similar to the general continuum equation (37) but with additional *conserved* KPZ nonlinearities [49]:

(50) $$\begin{array}{ccc} \frac{\partial h}{\partial t} & = & -v_{0}+\gamma \frac{\partial h}{\partial x}+\sum_{i=x,y}\left\{-\nu_{i}\frac{\partial^{2}h}{\partial i^{2}}+\zeta_{i}^{\left(1\right)}{\left(\frac{\partial h}{\partial i}\right)}^{2}+\Omega_{i}\frac{\partial^{2}}{\partial i^{2}}\frac{\partial }{\partial x}h\right.\\ & & \left.+\xi_{i}\left(\frac{\partial h}{\partial x}\right)\left(\frac{\partial^{2}h}{\partial i^{2}}\right)\right\}-\sum_{i,j=x,y}\left\{K_{ij}\frac{\partial^{2}}{\partial i^{2}}\frac{\partial^{2}}{\partial j^{2}}h+\zeta_{ij}^{\left(2\right)}\frac{\partial^{2}}{\partial i^{2}}{\left(\frac{\partial h}{\partial j}\right)}^{2}\right\}. \end{array}$$

The coefficients of the coupled two-field (C2F) model differ from those of the general equation and are given by [82]

(51) $$\begin{array}{ccc} \gamma & = & -\phi \alpha_{0}\alpha_{1x}, \end{array}$$

(52) $$\begin{array}{ccc} \nu_{x} & = & \phi \alpha_{0}\alpha_{2x}-\frac{\alpha_{0}^{2}}{\gamma_{0}}\bar{\phi }\phi \alpha_{1x}^{2}, \end{array}$$

(53) $$\begin{array}{ccc} \nu_{y} & = & \phi \alpha_{0}\alpha_{2y}, \end{array}$$

(54) $$\begin{array}{ccc} \zeta_{i}^{\left(1\right)} & = & -\phi \alpha_{0}\alpha_{6i}, \end{array}$$

(55) $$\begin{array}{ccc} \Omega_{i} & = & \alpha_{0}\left[-\phi \alpha_{3i}\left(\frac{\bar{\phi }K}{\gamma_{0}}-\phi R_{eq}\gamma_{2i}\right)\alpha_{1x}\right], \end{array}$$

(56) $$\begin{array}{ccc} \xi_{i} & = & \phi \alpha_{0}\alpha_{5i}, \end{array}$$

(57) $$\begin{array}{ccc} K_{ij} & = & KR_{eq}\gamma_{2i}+\alpha_{0}\left[\phi \alpha_{4ij}-\left(\frac{K\bar{\phi }}{\gamma_{0}}-\phi R_{eq}\gamma_{2i}\right)\alpha_{2j}\right], \end{array}$$

(58) $$\begin{array}{ccc} \zeta_{ij}^{\left(2\right)} & = & -\alpha_{0}\left(\frac{\bar{\phi }K}{\gamma_{0}}-\phi R_{eq}\gamma_{2i}\right)\alpha_{6j}. \end{array}$$

The main novelty of the C2F model is the incorporation of *redeposition* of eroded material to the surface with the parameter *ϕ* controlling the amount of redeposited atoms. A key feature of this model is the presence of ripple coarsening which is probably induced by the conserved KPZ nonlinearity [49,50,83]. Depending on the ratio between the coefficients of the conserved and the nonconserved KPZ terms, *i.e.,* $\zeta_{i}^{\left(1\right)}$ and $\zeta_{ij}^{\left(2\right)}$, very different time dependencies of the ripple wavelength have been observed, ranging from marginal logarithmic to strong power-law coarsening. Moreover, in agreement with some experiments [9,11,12,39,41,44,84], the observed coarsening is interrupted at a certain time and the wavelength saturates at a constant value [49,50].

## 3. Morphology of Ion-sputtered Si Surfaces

Because of its great technological relevance, e.g., in micro- and nanoelectronics, silicon has attracted considerable attention during the last decades as an interesting material for nanopatterning by ion erosion [3,5,9,11,14,38,42,43,72,85,86,87,88,89,90,91]. Thus, pattern formation on Si surfaces under various experimental conditions is well studied. However, the morphology of ion-sputtered Si surfaces exhibits some rather peculiar features and thus represents an interesting challenge for comparison with continuum theories. In this section, the morphology development of the Si surface during sub-keV ion sputtering will be summarized and discussed in the context of the different continuum models and in view of potential applications in thin film growth.

**Figure 5:**
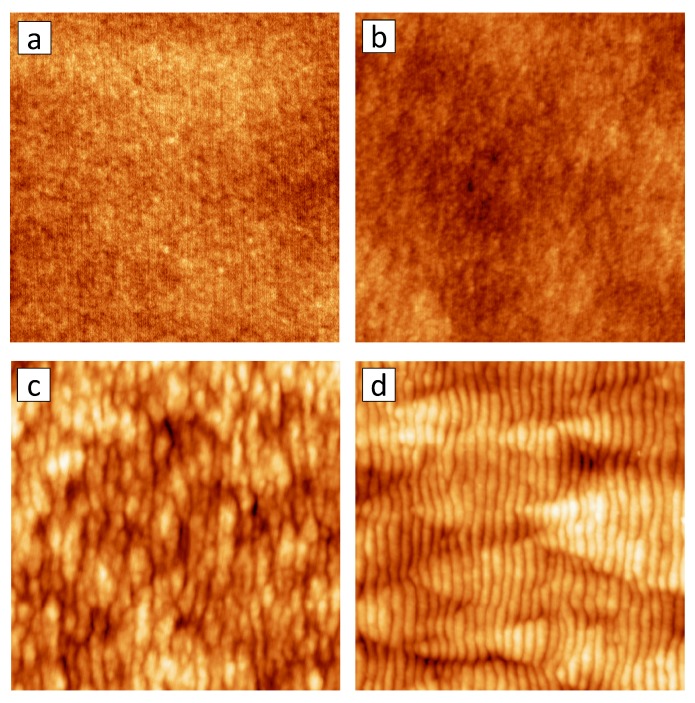
AFM images of Si surfaces after sputtering with 500 eV Ar ions under different angles *θ*: (a) $\theta =30^{\circ }$, $\Phi =5.7\times 10^{18}$ cm${}^{-2}$, height scale 2 nm; (b) $\theta =50^{\circ }$, $\Phi =2\times 10^{18}$ cm${}^{-2}$, height scale 3 nm; (c) $\theta =55^{\circ }$, $\Phi =2\times 10^{18}$ cm${}^{-2}$, height scale 3 nm; (d) $\theta =67^{\circ }$, $\Phi =1.7\times 10^{18}$ cm${}^{-2}$, height scale 10 nm. The size of the images is 1×1 *μ*m${}^{2}$; the ion beam was entering from the left.

### 3.1. The role of the incident angle: smoothing *vs.* roughening

Figure 5 depicts AFM images of Si surfaces sputtered with 500 eV Ar ions at room temperature and different incident angles *θ*. At rather low incident angles $\theta ≲50^{\circ }$ (Figure 5(a,b)), the Si surface remains flat. At a slightly larger incident angle of $\theta =55^{\circ }$ (Figure 5(c)), however, the formation of shallow and rather disordered ripples that are oriented normal to the direction of the ion beam is observed. The wavelength of these ripples is about 50 nm. A further increase of the incident angle to $\theta =67^{\circ }$ leads to a well ordered pattern of long homogeneous ripples with a periodicity of about 35 nm.

The observation that the Si surface remains flat at small incident angles is at variance with the BH model and most of its nonlinear extensions which predict an instability of the surface during ion sputtering independent of the experimental parameters. Carter and Vishnyakov explained a similar observation on Si surfaces bombarded with Xe ions of 10 to 40 keV energy as caused by an additional ion-induced mass transport along the surface that acts mainly at normal and near-normal incidence but is of minor importance at larger incident angles [9]. This so-called *ballistic diffusion* can also be introduced into the BH equation where it results in an additional smoothing term proportional to $\nabla^{2}h$ [9,92]. A similar mechanism has also been proposed for lower ion energies [92]. On the other hand, other experimental studies report dot and ripple pattern formation on Si surfaces also under normal and near-normal ion incidence [11,38,93,94]. However, recent experiments indicate that pattern formation under these low incidence conditions requires the presence of metal contaminations on the surface that may originate from the ion source [95] or the sample holder [96,97]. It has also been demonstrated that the resulting morphology of the Si surface can be tuned by varying the amount of metal contaminations during the sputtering [95,97]. A possible explanation for this so-called *seeding effect* invokes local variations of the sputter yield along the surface due to the segregation of deposited metal atoms that have a different component yield than Si [96]. A similar mechanism could also be responsible for the formation of dot patterns on compound semiconductors since there, preferential sputtering induces a form of "internal seeding" due to the enrichment and segregation of one atomic species on the surface. It has been shown theoretically that preferential sputtering can lead to a compositional modulation of the rippled surfaces of compound materials with the ripple crests having a different chemical composition than the valleys [98]. Since ion bombardment leads to an increase of the number of free bonds on the Si surface, also silicide formation could occur which would again alter the surface chemistry and thus also lead to a variation of the local sputter yield [96,99]. However, the presence of silicides on the sputtered Si surface could not be verified yet [95,96]. Also an increase of surface stress due to the seed atoms has been suggested as a possible origin of the dot patterns, a hypothesis that is supported by the experimental observation of tensile stress development in the presence of seeding [96,100].

With increasing angle of incidence, the BH model and the resulting linear and nonlinear continuum equations predict a rotation of the ripple pattern from normal to parallel with respect to the ion beam. Although this ripple rotation has been confirmed on various materials like metals [40,61], SiO${}_{2}$ [1,44,60,62], and graphite [36], the formation of ripple patterns oriented parallel to the direction of the ion beam at grazing incidence seems to be suppressed on Si surfaces at room temperature, so that only shallow anisotropic structures have been observed [101,102] that do not resemble the well ordered patterns obtained at elevated sample temperature [5,72]. However, recent experiments by Mollick and Ghose [103] showed that the formation of a clearly developed rotated ripple pattern under $80^{\circ }$ incidence can be induced also at room temperature by a chemical pre-roughening of the Si surface which is known to influence the dynamics of the pattern development [45,91].

### 3.2. Evolution of the surface morphology

The various continuum models discussed in section 2.3 make different predictions for the temporal evolution of the surface morphology especially in the limit of long times where nonlinearities dominate. Therefore, the fluence dependence of certain parameters that characterize the surface morphology, e.g., the ripple amplitude and wavelength, is of particular importance for identifying a potential continuum description of the given experimental system. In addition, as will be shown below, the ion fluence is also a crucial parameter for the optimization of the pattern quality which therefore directly affects possible applications of the nanopatterned surfaces. Thus, in this section, the morphology evolution of Si surfaces will be discussed in detail for the example of sub-keV sputtering under $67^{\circ }$ incidence. At this incident angle, the formed ripple patterns exhibit the highest quality, a fact that might be correlated with the maximum of the sputter yield in this angular region.

**Figure 6:**
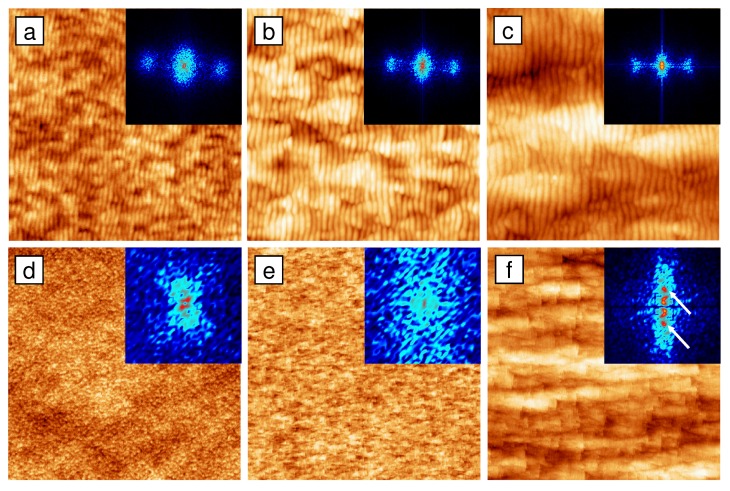
AFM images of Si(100) after sputtering with 300 eV ${\mathrm{Ar}}^{+}$ ions at fluences $\Phi =1\times 10^{17}$ (a,d), $5\times 10^{17}$ (b,e), and $1\times 10^{19}$ ${\mathrm{cm}}^{-2}$ (c,f). Height scales are 7 nm (a), 10 nm (b), 16 nm (c), 8 nm (d), 13 nm (e), and 28 nm (f). The size of the images is 1×1 *μ*m${}^{2}$ (a–c) and 5×5 *μ*m${}^{2}$ (d–f), respectively; the ion beam was entering from the left. Insets: corresponding FFT ranging from -75 to +75 $\mu m^{-1}$ (a–c) and from -4 to +4 $\mu m^{-1}$ (d–f) [39].

#### 3.2.1. Formation of two ripple modes

Figure 6(a–c) shows AFM images of the Si surface obtained after 300 eV bombardment with different fluences. At low fluence (Figure 6(a), $\Phi =1\times 10^{17}$ ${\mathrm{cm}}^{-2}$), the surface exhibits a pattern of shallow ripples oriented *normal* to the ion beam projection. In the following this pattern is called *normal pattern*. The two-dimensional Fourier transform (FFT) of this image (see inset of Figure 1(a)) shows two clearly separated side peaks. The position of the side peaks corresponds to the periodicity of the pattern, yielding a normal wavelength $\lambda_{n}∼20$ nm. With increasing fluence (Figure 6(b), $\Phi =5\times 10^{17}$ ${\mathrm{cm}}^{-2}$), corrugations overlay the normal pattern and get more pronounced until they become the dominating feature of the surface (Figure 6(c), $\Phi =1\times 10^{19}$ ${\mathrm{cm}}^{-2}$). At higher fluences, the surface reaches a steady state with reduced order and quality of the normal ripples.

Larger area AFM scans (Figure 6(d–f)) reveal that the corrugations overlaying the normal pattern become anisotropic with increasing fluence and finally form a quasi-periodic pattern at high fluences, which is oriented *parallel* to the beam direction (Figure 6(f)). This pattern is referred to as *parallel pattern*. Although the parallel pattern exhibits a much lower degree of order, side peaks can be identified (indicated by the white arrows) in the FFT, as shown in the inset of Figure 6(f). The side peaks indicate the quasi-periodicity of the parallel pattern and their position yields a much larger spatial periodicity of $\lambda_{p}∼900$ nm.

**Figure 7:**
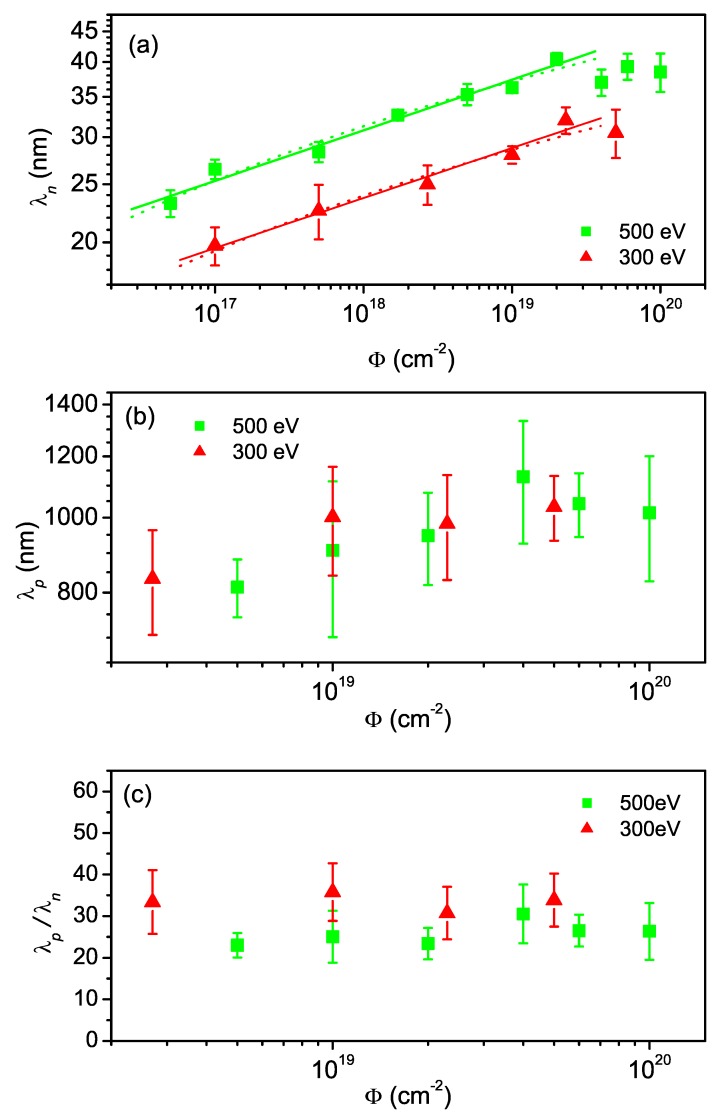
Evolution of (a) normal wavelength $\lambda_{n}$, (b) parallel periodicity $\lambda_{p}$, and (c) ratio of parallel to normal periodicity $\lambda_{p}/\lambda_{n}$ over fluence for 300 eV and 500 eV. The solid lines in (a) represent power law fits, yielding coarsening exponents of n=0.085 ± 0.006 and n=0.084 ± 0.007 for 500 eV and 300 eV, respectively. The dotted lines represent logarithmic fits [39].

In Figure 7(a) the fluence dependence of the normal wavelength $\lambda_{n}$, determined from the FFT of each AFM image, is depicted. Interrupted wavelength coarsening following a power law or logarithmic dependence is observed as soon as the ripple pattern is formed. Since wavelength coarsening is a nonlinear phenomenon, this indicates that nonlinearities start to dominate the surface evolution so early that no purely linear regime can be observed in the current experiments. In addition, $\lambda_{n}$ is found to increase with ion energy, indicating that ion-induced diffusion is the dominating smoothing process (cf. Section 2.3). This is also in agreement with the observed independence of $\lambda_{n}$ on the ion flux. The evolution of $\lambda_{p}$ is shown in Figure 7(b). Again, coarsening is observed. Figure 7(c) depicts the ratio of the wavelengths $\lambda_{p}/\lambda_{n}$. This ratio is quite constant in the investigated fluence range, indicating that both ripple modes exhibit similar coarsening behavior.

**Figure 8:**
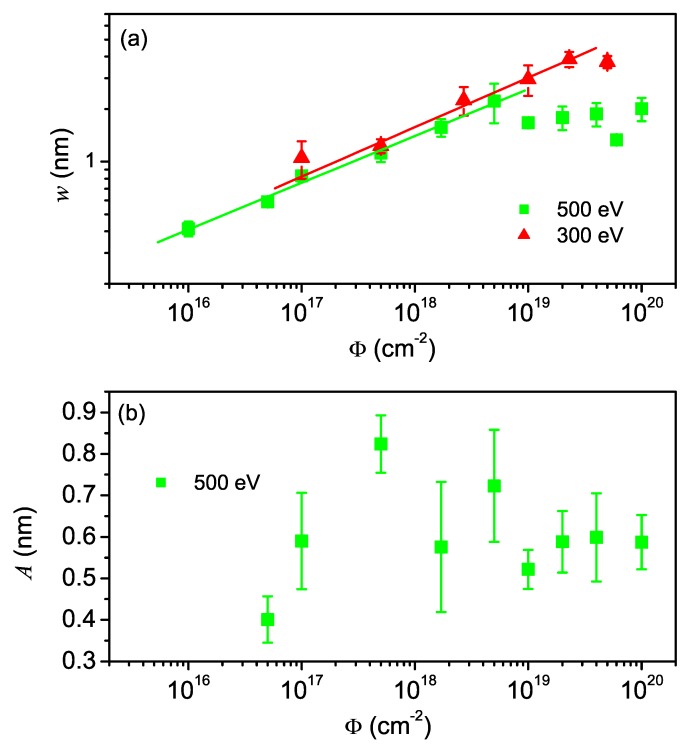
Surface roughness *w* (a) and ripple amplitude *A* (b) *versus* fluence. The solid lines in (a) represent power law fits, yielding growth exponents of β=0.28 ± 0.03 and β=0.27 ± 0.02 for 500 eV and 300 eV, respectively [39,104].

The evolution of the root-mean-square (rms) surface roughness *w* which describes the fluctuations of surface heights around the mean height and was calculated from the AFM images is shown in Figure 8. For both ion energies, *w* increases following a power law until it saturates at high fluences. One should note, however, that the rms roughness is not determined by the amplitude of the normal ripple pattern but rather by the larger corrugations and the parallel pattern, respectively. This is shown in Figure 8(b) that depicts the evolution of the ripple amplitude *A*, defined as the half of the average peak-to-peak height of the ripples, for the case of 500 eV sputtering. In the low fluence regime, the amplitude *A* is increasing from initially 0.4 nm to a maximum value of about 0.8 nm at $\Phi ≃5\times 10^{17}$ cm${}^{-2}$. For higher fluences, the amplitude decreases again and finally saturates at a value of $A^{sat}≃0.6$ nm. A similar overshooting before saturation has already been observed in previous experiments under normal ion incidence [37] and simulations of the anisotropic KS equation [34]. However, in contrast to the surface evolution in the KS equation, the experimentally observed saturation of the ripple amplitude is not accompanied by a loss of lateral order as is evident from Figure 6 which clearly shows a conservation of the pattern even at highest fluences. In combination with the observed interrupted wavelength coarsening, this suggests the C2F model as a potential description of the ripple formation and evolution on Si surfaces under these experimental conditions.

#### 3.2.2. Dynamic scaling behavior

In the C2F model, with the interruption of the coarsening the surface enters a long-time regime that exhibits kinetic roughening at large lateral scales and a preservation of the ripple pattern at small scales [83]. Such a behavior is also seen in the experimental results presented in Figure 6. A kinetically rough surface is invariant under appropriate rescaling of its lateral and vertical dimensions and the time *t* [74]. This results in a certain behavior of its surface roughness $w\left(l,t\right)={\langle {\left[h\left(\vec{r},t\right)-{\langle h\left(\vec{r},t\right)\rangle }_{l}\right]}^{2}\rangle }_{l}^{1/2}$ where $h(\vec{r},t)$ is the surface height function, *l* is the size of the observation window over which *w* has been calculated, and the angular brackets denote spatial averaging. In the case of Family-Vicsek (FV) dynamic scaling [105], the roughness should scale as $w\left(l,t\right)∼t^{\beta }$ until the correlation length $\xi \left(t\right)∼t^{1/z}$ has reached the window size *l*. Then, the roughness will saturate with the saturation value depending on the window size, $w\left(l\right)∼l^{\alpha }$. The roughness exponent *α*, the growth exponent *β*, and the dynamic exponent z=α/β characterize the surface in space and time and can be used to attribute the system to a certain universality class and, therefore, to a certain continuum equation [74]. With this intention, the dynamic scaling behavior of the ion-sputtered Si surface has been analyzed by evaluating its one-dimensional structure factor. According to the dynamic scaling hypothesis [74], the one-dimensional structure factor should obey the relation

(59) $$S\left(k,t\right)=k^{-(2\alpha +1)}s\left(kt^{1/z}\right)$$

with the scaling function $s\left(u\right)∼u^{2\alpha +1}$ and s(u)∼ const. for u≪1 and u≫1, respectively. In the case of anisotropic surfaces, this behavior is modified and the surface is characterized in the normal and parallel direction in real and momentum space by four different roughness exponents [106]. However, for $kt^{1/z}\gg 1$, the dynamic scaling behavior of the one-dimensional structure factor can still be described by Equation (59) [104,106].

**Figure 9:**
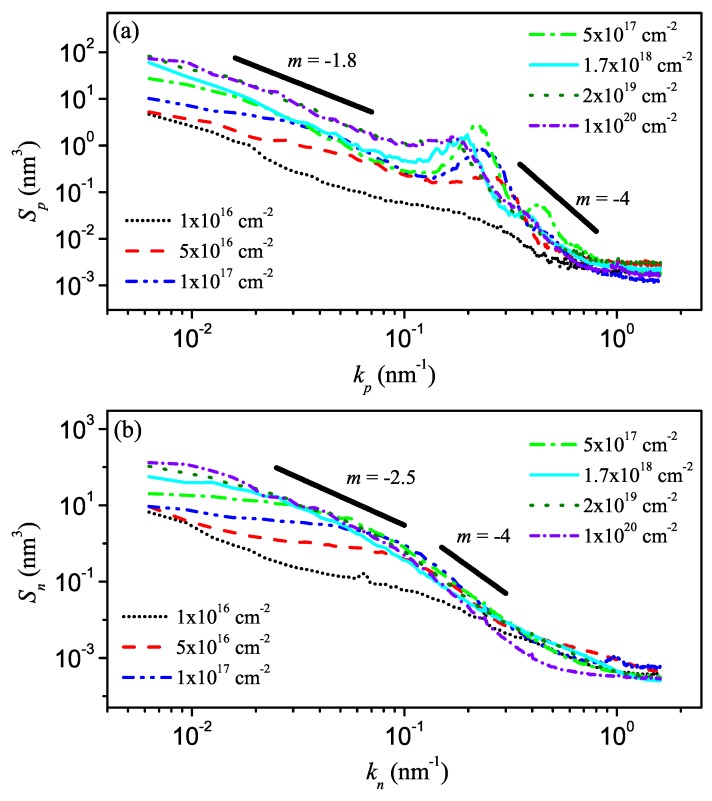
Structure factors $S_{p,n}\left(k_{p,n}\right)$ in the direction parallel (a) and normal (b) to the ion beam at different fluences. The solid straight lines correspond to $S_{p,n}∼k_{p,n}^{m}$ [104].

The structure factor $S_{p}\left(k_{p}\right)$ calculated in the direction parallel to the ion beam is given in Figure 9(a). For $\Phi \geq 5\times 10^{16}$ cm${}^{-2}$, a peak appears at the spatial frequency $k_{p}^{*}$ corresponding to the wavelength *λ* of the ripple pattern. For $k_{p}\gg k_{p}^{*}$, the $S_{p}$ curves all collapse. The slope *m* (in the log-log plot) of the curves in this regime is about -4, corresponding to a roughness exponent of 1.5. With increasing fluence, the ripples coarsen and the position of the peak is shifting to smaller $k_{p}$ values. Also the structure factor increases with fluence for $k_{p}\ll k_{p}^{*}$ and a second scaling regime develops at high fluences. Here, the roughness exponent was determined to be $\alpha_{p}=0.41\pm 0.04$. In Figure 9(b), the structure factor curves in the direction normal to the ion beam, $S_{n}\left(k_{n}\right)$, are depicted for different fluences. At large values of $k_{n}$, the data is consistent with a slope m=-4. At small $k_{n}$ values, $S_{n}\left(k_{n}\right)$ again increases with fluence and until a power-law behavior with a roughness exponent $\alpha_{n}=0.76\pm 0.04$ appears at high fluences.

The observed peak in the structure factor $S_{p}$ in the direction parallel to the ion beam with the -4 slope at large $k_{p}$ values (cf. Figure 9(a)) indicates the presence of a KS like instability in this direction [35]. The orientation of the ripples with respect to the incident ion beam is determined by the signs of the linear coefficients: the wave vector of the observed ripple structure is parallel to the direction with the smallest negative *ν* (cf. section 2.2). Therefore, for the here presented experiment $\nu_{p}<\nu_{n}$. In the direction normal to the ion beam, the experimental $S_{n}$ curves shown in Figure 9(b) do not exhibit a local maximum. The determined low-fluence behavior for the *n* direction $S_{n}\left(k_{n}\right)∼k_{n}^{-4}$ corresponds to the scaling behavior of the one-dimensional linear molecular beam epitaxy (lMBE) equation with $\alpha_{lMBE}=3/2$ [74]. This indicates that the very short-distance behavior of the sputtered Si surface is dominated by the diffusion term. This behavior holds even at the highest applied fluence of $\Phi =1\times 10^{20}$ cm${}^{-2}$ without any noticeable crossover. This indicates that $|\nu_{n}|\approx 0$ [104].

In the limit of high fluences $\Phi \geq 10^{19}$ cm${}^{-2}$, the morphology of the Si surface exhibits anisotropic algebraic scaling at large lateral scales with $\alpha_{n}=0.76$ and $\alpha_{p}=0.41$. The KS equation (31) is not able to reproduce such an anisotropic scaling behavior since the only term breaking the x→-x symmetry is the one with the coefficient *γ* which acts only at rather short length scales. On the other hand, the dispersive nonlinearities with the coefficients $\xi_{x,y}$ that appear both in the general continuum equation (37) and in the C2F model (50) have been found to induce anisotropic scaling under certain conditions [106]. Therefore, the appearance of anisotropic scaling supports above assumption of the C2F model being a suitable description of the Si surface during sub-keV ion sputtering [104].

#### 3.2.3. Dynamics of topological pattern defects

In view of possible applications of the nanorippled Si surfaces, the appearance of kinetic roughening, *i.e.,* of a disordered state, at high fluences is not favorable since most of these applications rely on a well-ordered homogeneous pattern. Therefore, the applied ion fluence is an important parameter in the fabrication of nanopatterned surfaces and vital for optimizing their quality.

**Figure 10:**
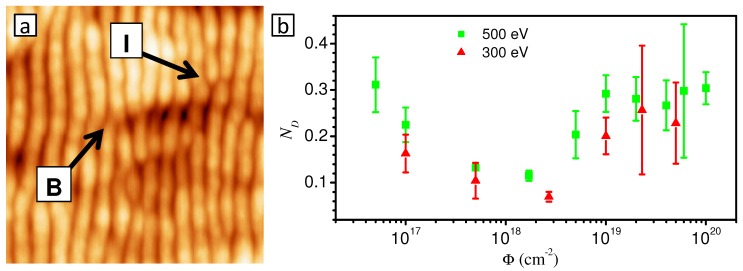
(a) AFM image of a rippled Si surface with a bifurcation (B) and an interstital (I). The size of the image is 400×400 nm${}^{2}$. (b) Evolution of the normalized density of pattern defects $N_{D}$ for 500 and 300 eV Ar sputtering of Si under $67^{\circ }$ incidence [107].

The quality of the ripple patterns can be quantified by calculating a normalized density of topological pattern defects from the AFM images [86,103,107]. In this context, topological pattern defects means either a bifurcation (B) of a ripple, *i.e.,* a Y junction of ripples, or an interstitial (I), *i.e.,* a discontinuous or broken ripple. Figure 10(a) shows an AFM image of the rippled Si surface in which these defect types are indicated.

**Figure 11:**
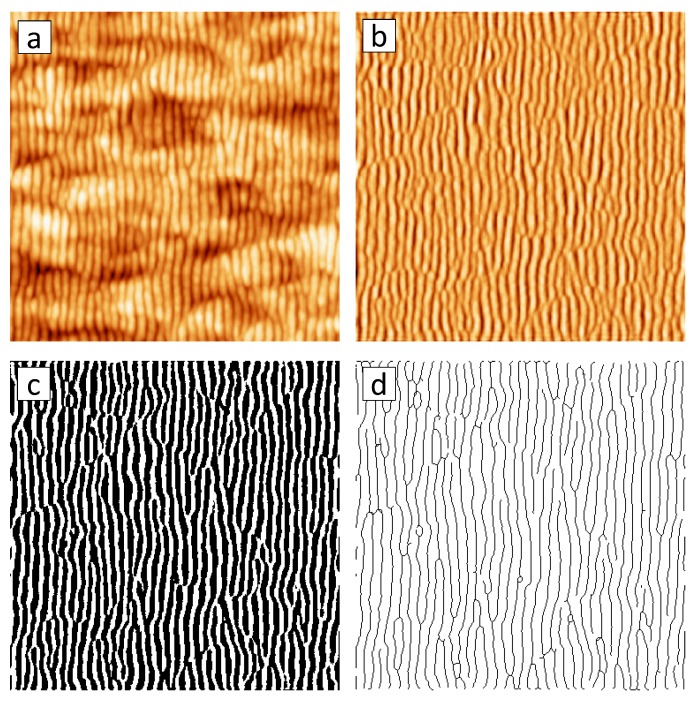
AFM image (a) after Fourier-filtering (b), conversion into a binary image (c) and thinning the ripples to single pixel lines (d). The size of the images is 1×1 *μ*m${}^{2}$.

The procedure of determining the normalized density of topological pattern defects is demonstrated in Figure 11. In order to determine the total number of defects of a given AFM image (Figure 11(a)), the image is Fourier-filtered to remove the long-wavelength surface morphology (Figure 11(b)). The filtered image is then converted into a binary image by applying Otsu’s threshold [108] (Figure 11(c)). Finally, the ripples in the binary image are thinned to lines of one pixel width (Figure 11(d)). Then, every black pixel with more or less than two black neighboring pixels is counted as a defect. The normalized density of defects is then calculated as $N_{D}=N\lambda^{2}/A_{S}$ with the total number of defects *N* of the image, the ripple periodicity *λ*, and the scan area $A_{S}$. A value of $N_{D}=0$ then corresponds to a perfect pattern without any defects and $N_{D}=1$ to a pattern in which each ripple contains one defect per length *λ*.

Following this approach, the normalized density of pattern defects $N_{D}$ has been calculated for different fluences in order to monitor the evolution of the pattern quality. The result is shown in Figure 10(b). The $N_{D}$ values are comparable for both energies, although in average $N_{D}$ appears to be slightly lower for 300 eV than for 500 eV. At the lowest fluence $\Phi =5\times 10^{16}$ cm${}^{-2}$, the normalized defect density is around 0.3. With increasing fluence, $N_{D}$ decreases until it reaches a minimum value of $N_{D}∼0.1$ (500 eV) and 0.07 (300 eV) at a fluence of $\Phi ∼2\times 10^{18}$ cm${}^{-2}$. This decrease of $N_{D}$ is caused by the growth of the ripple length and the annihilation of pattern defects due to a complex interplay of different "annealing" processes [107]. At higher fluences, $N_{D}$ increases again until it saturates at $\Phi ∼10^{19}$ cm${}^{-2}$ at a value of $N_{D}∼0.28$. This increase results from the appearance of kinetic roughening which induces a certain disorder in the pattern that leads to the formation of "defect clusters" [107]. Interestingly, the coarsening of the ripple wavelength does not seem to be related to the evolution of the pattern defects (cf. Figure 7(a) and Figure 10(b)). This is in contrast to other experimental systems like Pt(111) surfaces under grazing incidence sputtering where rapid coarsening proceeds due to the annihilation of defects [109].

These results demonstrate the influence of the applied fluence not only on the ripple amplitude and wavelength but also on the pattern quality. Therefore, in order to fabricate patterns of a certain periodicity in the highest quality possible, the interplay between fluence, energy, pattern wavelength, and pattern quality needs to be known.

## 4. Summary

We have presented an overview of the continuum approach to ion-induced pattern formation on amorphous surfaces. The predictions of the various linear and nonlinear continuum models have been discussed and compared to experimental observations with a special focus on sub-keV ion sputtering of Si surfaces. Because of its potential applications, pattern formation on Si surfaces induced by low-energy sputtering has been investigated excessively during the last two decades. These studies revealed several peculiarities of the morphology of sputtered Si surfaces such as the stability of the flat surface at near-normal incidence, (interrupted) wavelength coarsening or the absence of a pattern rotation with increasing angle of incidence. In addition, contradictory observations have been reported, e.g., the occurrence of smoothing and roughening at small incident angles, respectively.

Recent experimental findings such as the importance of metal contaminations during the sputtering, delivered further insight into the basic mechanisms of ion-induced pattern formation on Si surfaces. In addition, novel and elaborated theoretical models provided new explanations for certain experimental observations, e.g., wavelength coarsening or the occurrence of anisotropic scaling. Therefore, a rather coherent picture of the morphology of ion-sputtered Si surfaces has developed during the last few years. However, at the same time new challenges, both experimental and theoretical ones, have appeared, among them the control of surface contaminations and the investigation of its detailed effects on the morphology development which might enable the fabrication of novel nanopatterned surfaces.

On the other hand, the application of nanorippled Si substrates in various fields of modern materials science, especially in nanoscale magnetism and plasmonics, is developing tremendously and demands for a precise control over the fabricated patterns. Besides the tuning of the wavelength and the amplitude, the quality, order, and regularity of the patterns is becoming more and more important since the order has a strong effect on the degree of the induced functional anisotropy. Providing nanopatterned substrates of high quality and with tailored properties has thus become a major experimental issue.
